# Contemporary practice pattern of permanent pacing for conduction disorders in inferior ST‐elevation myocardial infarction

**DOI:** 10.1002/clc.23210

**Published:** 2019-06-07

**Authors:** Naoki Misumida, Gbolahan O. Ogunbayo, John Catanzaro, Farshid Etaee, Sun Moon Kim, Ahmed Abdel‐Latif, Khaled M. Ziada, Claude S. Elayi

**Affiliations:** ^1^ Gill Heart and Vascular Institute and Division of Cardiovascular Medicine University of Kentucky Lexington Kentucky; ^2^ Department of Cardiology University of Texas Southwestern Medical Center Dallas Texas; ^3^ Devision of Cardiovascular Medicine University of Florida Jacksonville Florida

**Keywords:** complete atrioventricular block, high‐degree atrioventricular block, pacemaker, sinoatrial node dysfunction, ST‐elevation myocardial infarction

## Abstract

**Background:**

Currently, there is no clear consensus regarding the optimal waiting period before permanent pacemaker implantation in patients with conduction disorders following an inferior myocardial infarction.

**Hypothesis:**

We aimed to elucidate the contemporary practice pattern of pacing, especially the timing of pacemaker implantation, for sinoatrial node and atrioventricular (AV) conduction disorders following an inferior ST‐elevation myocardial infarction (STEMI).

**Methods:**

Using the National Inpatient Sample database from 2010 to 2014, we identified patients with a primary diagnosis of inferior STEMI. Primary conduction disorders were classified into: (a) high‐degree AV block (HDAVB) consisting of complete AV block or Mobitz‐type II second‐degree AV block, (b) sinoatrial node dysfunction (SND), and (c) no major conduction disorders.

**Results:**

Among 66 961 patients, 2706 patients (4.0%) had HDAVB, which mostly consisted of complete AV block (2594 patients). SND was observed in 393 patients (0.6%). Among the 2706 patients with HDAVB, 267 patients (9.9%) underwent permanent pacemaker. In patients with HDAVB, more than one‐third (34.9%) of permanent pacemakers were placed within 72 hours after admission. The median interval from admission to permanent pacemaker implantation was 3 days (interquartile range; 2‐5 days) for HDAVB vs 4 days (3‐6 days) for SND (*P* < .001). HDAVB was associated with increased in‐hospital mortality, whereas SND was not.

**Conclusions:**

In patients who developed HDAVB following an inferior STEMI, only one in 10 patients underwent permanent pacemaker implantation. Despite its highly reversible nature, permanent pacemakers were implanted relatively early.

## 1 INTRODUCTION

Sinoatrial node and atrioventricular (AV) conduction disorders often complicate inferior myocardial infarction immediately to a few days following the initial ischemic event.^1^, ^2^, ^3^, ^4^ Among the potential conduction disorders, high‐degree AV block (HDAVB), which includes complete AV block and Mobitz‐type II second‐degree AV block have been the primary focus of prior studies. In these studies, HDAVB after an inferior myocardial infarction was associated with increased short‐term mortality, but resolved spontaneously in the vast majority of patients in the thrombolytic and primary percutaneous coronary intervention (PCI) era.^5^, ^6^, ^7^, ^8^, ^9^

In view of its highly reversible nature, clinicians often face a dilemma with regards to the optimal timing of permanent pacemaker implantation for HDAVB following an inferior myocardial infarction. Although earlier implantation (a shorter waiting period) will facilitate recovery from the event and shorten the length of stay, both short‐ and long‐term complications related to permanent pacemaker are relatively common, affecting nearly 1 in 6 patients by 3 years and contributing to incremental healthcare cost.^10^ Currently, there is no clear consensus or recommendation regarding the optimal waiting period before permanent pacemaker implantation in patients with conduction disorders following an inferior myocardial infarction. In this context, we aimed to clarify the contemporary practice pattern of pacemaker implantation, especially the timing of implantation, in patients with inferior ST‐elevation myocardial infarction (STEMI) using a large national inpatient database. We also aimed to investigate the incidence and prognostic impact of sinoatrial node dysfunction (SND), another well‐known conduction disorder associated with inferior myocardial infarction.^3^, ^4^, ^11^, ^12^

## 2 METHODS

### 2.1 Data source

The National Inpatient Sample (NIS) database is the largest publicly available all‐payer inpatient care database in the United States, which is a part of the Healthcare Cost and Utilization Project from the Agency for Healthcare Research and Quality. This NIS dataset contains over 100 clinical and non‐clinical data elements from more than 7 million hospital discharges per year. The NIS dataset in the year 2010 and 2011 were sampled from all discharges from a stratified sample of approximately 20% of US community hospitals, whereas the dataset in the year 2012 to 2014 were sampled from a stratified sample of approximately 20% of discharges from all US community hospitals. The University of Kentucky Institutional Review Board deemed this study exempt from a formal review as the NIS database is available to the public as aggregate data without direct personal identifiers.^13^

### 2.2 Inclusion and exclusion criteria

We analyzed the NIS database from 2010 to 2014 and identified all hospitalized patients 18 years or older with a primary diagnosis of inferior STEMI using the International Classification of Diseases, Ninth Edition, Clinical Modification (ICD‐9‐CM) diagnosis codes 410.2x, 410.3x, and 410.4x. We only retained patients with the primary diagnoses of inferior STEMI as the main reason for the hospital admission. We excluded patients who were admitted and discharged alive on the same day and those transferred from outside hospitals to avoid duplicating records in line with a prior study from the NIS database on STEMI patients.^14^ Exclusion of transferred cases was also necessary to accurately evaluate the timing of pacemaker implantation. Lastly, we excluded patients with a prior pacemaker (ICD‐9‐CM code V45.01) or implantable cardioverter defibrillator implantation (ICD‐9‐CM code V45.02) in line with a prior study from NIS database.^9^

### 2.3 Diagnosis of conduction disorder and outcomes of interest

Patients' primary conduction disorder were hierarchically classified into three mutually exclusive categories in the following order: (a) HDAVB consisting of complete AV block (code 426.0) or Mobitz‐type II second‐degree AV block (code 426.12), (b) SND (code 427.81), and (c) no major conduction disorders for the remaining patients without HDAVB or SND.

Patients' baseline demographics and clinically relevant comorbidities were obtained using ICD‐9‐CM codes and the Elixhauser Comorbidity adjustment method.^15^ Secondary diagnoses and procedures performed during hospitalization were identified using ICD‐9‐CM codes and Clinical Classification Software codes. The primary outcomes of interest were the rate of permanent pacemaker implantation and its timing of implantation. The secondary outcomes included in‐hospital all‐cause mortality and hospital length of stay. A list of codes used to identify comorbidities and other outcomes is presented in Table S1.

### 2.4 Statistical analysis

Patients' characteristics, rate, and timing of permanent pacemaker implantation, and clinical outcomes were compared among patients with HDAVB, SND, and those without a major conduction disorder. Subgroup analysis was performed in the patients who underwent PCI (excluding patients who were medically managed and those who underwent coronary artery bypass grafting). Continuous data were expressed as mean ± SD or median with interquartile range (IQR) and were compared using Kruskal‐Wallis test and Mann‐Whitney *U* test as appropriate. Categorical variables were expressed in percentage and compared using the Pearson *χ*² test. Multivariable logistic regression models were constructed to identify predictors of HDAVB and SND. All patients' demographics, comorbidities, and hospital characteristics (shown in Table 1) were included in these models except for race, which was missing in 7.8% cases. Another multivariable logistic regression model was constructed to evaluate the prognostic impact of HDAVB and SND. In‐hospital revascularization procedures (PCI and coronary artery bypass grafting) as well as aforementioned patients and hospital characteristics were included in this model. To account for a large sample size and multiple comparisons, the type I error *α* was set to a more conservative value of <0.001; *P*‐values <.001 were considered statistically significant. All statistical analyses were performed with SPSS version 24.0 (IBM Corp, Armonk, New York).

**Table 1:** Demographics, risk factors, and hospital characteristics

	High‐degree AV block (n = 2706)	Sinoatrial node dysfunction (n = 393)	No major conduction disorder (n = 63 862)	*P*‐value
Age (mean ± SD)	67 ± 13^a^	74 ± 12^b^	62 ± 13^c^	<.001
Male	60.8%^a^	60.6%^a^	69.6%^b^	<.001
Race White	71.5%^a^	76.3%^a^	71.8%^a^	0.13
Hypertension	63.2%^a^	69.7%^a^	65.3%^a^	.02
Diabetes	33.2%^a^	33.1%^a,b^	27.6%^b^	<.001
Dyslipidemia	56.1%^a^	61.3%^a,b^	64.9%^b^	<.001
Current smoking	32.2%^a^	22.9%^b^	36.6%^c^	<.001
Obesity	12.6%^a^	13.2%^a^	14.1%^a^	.08
Coronary artery disease	15.8%^a^	11.5%^a,b^	13.0%^b^	<.001
Prior myocardial infarction	7.3%^a^	9.2%^a^	8.8%^a^	.02
Prior PCI	10.2%^a^	10.7%^a,b^	12.3%^b^	.003
Prior CABG	3.5%^a^	5.3%^a^	3.3%^a^	.06
Prior stroke/TIA	4.8%^a^	5.9%^a^	4.0%^a^	.03
Congestive heart failure	22.5%^a^	27.5%^a^	12.1%^b^	<.001
Anemia	16.7%^a^	23.2%^a^	9.9%^b^	<.001
Renal failure	13.1%^a^	22.9%^b^	8.1%^c^	<.001
Chronic pulmonary disease	17.1%^a^	19.1%^a,b^	14.7%^b^	<.001
Peripheral vascular disease	10.5%^a^	16.5%^b^	7.8%^c^	<.001
Atrial fibrillation or atrial flutter	18.3%^a^	45.0%^b^	11.2%^c^	<.001
Hospital location/ status	^a^	^a,b^	^b^	.007
Rural hospital	7.3%	7.4%	7.7%	
Urban teaching hospital	53.3%	47.9%	49.7%	
Urban nonteaching hospital	39.4%	44.6%	42.6%	
Hospital size	^a^	^a^	^a^	.69
Small	9.7%	7.7%	9.4%	
Medium	23.7%	25.1%	24.4%	
Large	66.6%	67.2%	66.2%	
Hospital region	^a^	^a^	^a^	.96
Midwest	22.6%	24.2%	23.1%	
Northeast	16.9%	16.3%	16.2%	
South	39.5%	39.2%	39.6%	
West	21.0%	20.4%	21.1%	
Cardiac arrest	20.7%^a^	14.5%^a^	8.5%^b^	<.001
Cardiogenic shock	34.0%^a^	15.8%^b^	8.0%^c^	<.001
In‐hospital PCI	84.7%^a^	74.8%^b^	83.5%^a^	<.001
In‐hospital CABG	5.1%^a^	6.6%^a^	5.2%^a^	.44
Length of stay (days; median [IQR])	4 [3–7]^a^	6 [3–9]^b^	3 [2–4]^c^	<.001
Cost ($; median [IQR])	85 342 [57235‐135 494]^a^	96 192 [59578‐151 647]^a^	64 994 [44400‐97 697]^b^	<.001

Abbreviations: AV, atrioventricular; CABG, coronary artery bypass grafting; IQR, interquartile range; PCI, percutaneous coronary intervention; TIA, transient ischemic attack.

Different alphabet (a, b, or c) denotes significant difference (*P* < .001).

## 3 RESULTS

### 3.1 Incidence of HDAVB and SND following inferior STEMI

**Figure 1:**
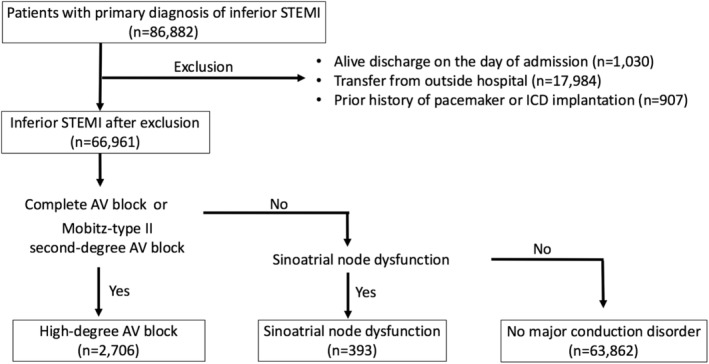
Flow chart of patient selection and diagnosis. ICD; implantable cardioverter defibrillator; STEMI, ST‐elevation myocardial infarction

After exclusions, we identified 66 961 patients (discharges) with a primary diagnosis of inferior STEMI. Patients' selection flow and hierarchical diagnostic flow chart are presented in Figure 1. A total of 2706 patients (4.0%) had HDAVB, which mostly consisted of complete AV block (2594 patients). SND was less prevalent than HDAVB and observed in 393 patients (0.6%). In the subgroup of the patients who underwent PCI (81.3% of the overall population), these incidences were essentially unchanged (4.1% for HDAVB and 0.5% for SND).

### 3.2 Characteristics of the patients with HDAVB and SND

Patients' characteristics are shown in Table 1. Patients with SND were older compared to other groups with a mean age of 74 years. Patients with HDAVB had a higher incidence of cardiogenic shock compared to other groups. Almost one‐half of the patients with SND (45.0%) had atrial fibrillation or atrial flutter. Independent predictors for HDAVB and SND are shown in Table 2. Independent predictors for HDAVB included age, female sex, diabetes, congestive heart failure, anemia, and atrial fibrillation or atrial flutter. Age and atrial fibrillation or atrial flutter were independent predictors for SND.

**Table 2:** Independent predictors for high‐degree atrioventricular (AV) block and sinoatrial node dysfunction

	Odds ratio	99% confidence interval	*P*‐value
High‐degree AV block			
Age (per decade increase)	1.20	1.14‐1.26	<.001
Female	1.19	1.07‐1.33	<.001
Hypertension	0.85	0.76‐0.95	<.001
Diabetes	1.29	1.15‐1.45	<.001
Dyslipidemia	0.77	0.69‐0.86	<.001
Congestive heart failure	1.54	1.35‐1.76	<.001
Anemia	1.33	1.15‐1.54	<.001
Atrial fibrillation or atrial flutter	1.27	1.11‐1.47	<.001
Sinoatrial node dysfunction			
Age (per decade increase)	1.69	1.48‐1.93	<.001
Atrial fibrillation or atrial flutter	3.77	2.84‐5.00	<.001

### 3.3 Temporary pacemaker use for HDAVB and SND

Among the 2706 patients with HDAVB, 885 patients (32.7%) had a temporary pacemaker and 88 patients of them subsequently underwent permanent pacemaker implantation. Most of the temporary pacemakers (85.2%) were inserted on the day of admission (day 0). Among the 393 patients with SND, 54 patients (13.7%) had a temporary pacemaker.

### 3.4 Rate of permanent pacemaker implantation for HDAVB and SND

Among the 2706 patients with HDAVB, 267 patients (9.9%) underwent permanent pacemaker, whereas the patients with SND were more likely to undergo permanent pacemaker implantation compared to those with HDAVB (37.4% [147/393]; *P* < .001 compared to HDAVB). This difference remained significant after excluding patients who died (39.0% vs 10.4%; *P* < .001). In the subgroup of the patients who underwent PCI, 8.0% of patients with HDAVB and 38.5% of those with SND underwent permanent pacemaker implantation.

### 3.5 Timing of permanent pacemaker implantation

**Figure 2:**
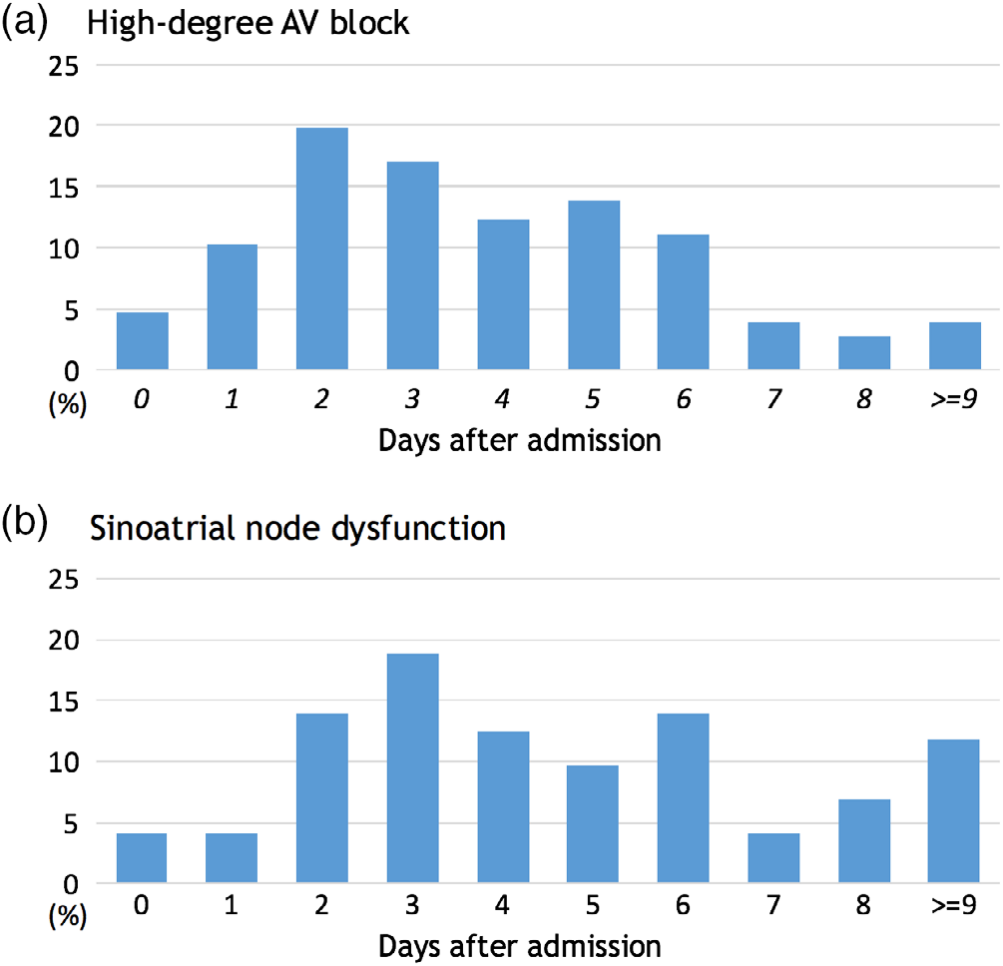
Timings of permanent pacemaker implantation: A, permanent pacemaker implantation for high‐degree atrioventricular block and B, permanent pacemaker implantation for sinoatrial node dysfunction

Figure 2 illustrates the timing of permanent pacemaker implantation. In patients with HDAVB, the median interval from admission to permanent pacemaker implantation was 3 days (IQR; 2‐5 days), more than one‐third (34.9%) of permanent pacemakers were implanted within 72 hours after admission (day 0, 1, or 2) and most permanent pacemakers (89.3%) were implanted by the end of day 6. In patients with SND, the median interval from admission to permanent pacemaker implantation was 4 days (IQR; 3‐6 days) which was longer than in HDAVB (*P* = .001). In the subgroup of the patients who underwent PCI, the median intervals from admission to permanent pacemaker were essentially unchanged (3 days [2‐5 days] for HDAVB vs 4 days [3‐6 days] for SND).

### 3.6 In‐hospital mortality in patients with HDAVB and SND

Patients with HDAVB had a significantly higher in‐hospital mortality than those without a major conduction disorder (13.1% vs 5.2%; *P* < .001). There was no statistically significant difference in in‐hospital mortality between patients with SND and those without conduction disorder (6.1% vs 5.2%; *P* = 0.39). Among patients who underwent permanent pacemaker implantation for HDAVB or SND, 6.7% of them died during hospitalization. Multivariable logistic regression analysis showed that HDAVB was an independent predictor for higher in‐hospital mortality (odds ratio 2.21; 99% confidence interval 1.87‐2.62; *P* < 0.001), whereas SND was not. Patients with HDAVB or SND had a longer hospital stay compared to those without a major conduction disorder (Table 1).

## 4 DISCUSSION

The main findings of our study include (a) in patients with inferior STEMI, HDAVB, and SND were observed in 4.0% and 0.6% of the patients, respectively, (b) less than 10% of the patients with HDAVB underwent permanent pacemaker implantation, and more than one‐third of those were implanted within 72 hours after admission (the median interval of 3 days since admission), and 3) HDAVB was associated with a higher in‐hospital mortality, whereas SND was not.

### 4.1 High‐degree AV block following inferior STEMI

HDAVB was observed in 4.0% of our cohort, which is consistent with the incidence previously reported in the primary PCI era.^8^ The predictors of HDAVB in our analysis were also similar to prior observations.^6^ Notably, less than 10% of the patients with HDAVB underwent permanent pacemaker implantation, similar to the 9% reported in another study.^8^ As persistent HDAVB usually requires pacemaker regardless of symptoms,^16^ this low rate of pacemaker implantation reflects the high reversibility of HDAVB with inferior STEMI. HDAVB is also strongly associated with cardiogenic shock, either as a cause or consequence, and the association with higher mortality during the acute or subacute phase of inferior myocardial infarction has been well‐documented.^5^, ^6^, ^8^, ^9^ Survival after discharge, however, seems comparable between those with and without HDAVB.^7^, ^8^, ^17^, ^18^

### 4.2 Timing of permanent pacemaker implantation for high‐degree AV block following inferior STEMI

In patients with inferior STEMI with subsequent conduction disorders, the decision whether to implant a permanent pacemaker is often not straightforward. Current guidelines recommend permanent pacemakers only for “persistent” infra‐nodal or symptomatic second‐ or third‐degree AV block (Class I or IIa indication), while “transient” AV block is a contraindication for pacemaker implantation (Class III).^16^ However, the difference between “persistent” and “transient,” which would translate into Class I or Class III indication, respectively, is often difficult to appreciate during the early phases of myocardial infarction. Thus, we frequently face a clinical dilemma regarding the optimal period of observation required to establish the “persistence” of an AV block with a degree of certainty. Prior reports demonstrate it may take up to 2 weeks for HDAVB to recover.^19^ The optimal period of observation before pacemaker implantation has been long debated.^20^ In 1997, after reviewing available literature in the pre‐thrombolysis and thrombolysis era, Barold concluded that “there is no need to rush insertion of a permanent pacemaker in AV block secondary to inferior MI.”^20^

In the contemporary era of primary PCI, the optimal period of observation after an inferior MI for pacemaker implantation remains to be established. In our cohort, about one‐third of permanent pacemakers were implanted within 72 hours after admission. This short waiting period raises the question of how many permanent pacemaker implantations could have been avoided if there was sufficient time for HDAVB to recover or self‐resolve. A recent study revealed that short‐ and long‐term complications related to permanent pacemaker are relatively common, affecting nearly 1 in 6 patients by 3 years and contributing to considerable incremental health care cost.^10^ Nevertheless, a longer waiting period is not without risks. This can be associated with the risk of iatrogenic complications, such as infections with the use of temporary pacemakers, deep venous thrombosis due to immobility, deconditioning especially in elderly patients, and increased length of stay and costs. Overall, there are advantages and disadvantages of early vs later pacemaker implantation after an inferior STEMI and HDAVB. In our opinion, however, waiting a few days prior to device implantation is in general the preferable option because the majority of patients will not require permanent pacing.

### 4.3 Sinoatrial node dysfunction following inferior STEMI

Although SND has been established as one of the conduction disorders associated with inferior myocardial infarction, it seems SND has received much less attention compared to HDAVB probably due to its low incidence.^3^, ^4^, ^11^ SND can occur very early and transiently, in the first few minutes of an acute inferior myocardial infarction, often due to the vagal Bezold‐Jarish reflex.^21^, ^22^ This early form of SND, which typically responds to atropine, often resolves within the first few hours and mostly within 24 hours.^19^ Therefore, SND may only be observed in patients by paramedics or while in an emergency room. In our contemporary cohort, the incidence of SND during hospitalization was 0.6%, which is lower than the findings from previous studies conducted during the pre‐thrombolysis era.^3^, ^4^ SND that persists beyond the first few hours is more likely to be captured as an in‐hospital diagnosis and therefore our incidence likely represents more persistent and/or clinically significant SND.

In our cohort, SND was much less common than HDAVB, but more likely to lead to permanent pacemaker implantation. One explanation for the high rate of pacemaker implantation in SND is the possible worsening of pre‐existing or underlying conduction abnormalities. When patients develop inferior STEMI, they are exposed to a variety of factors that can unmask or worsen SND, including vagal reflex, ischemia of the sinus node, electrolytes imbalances, and medications such as beta‐blockers.^11^, ^23^, ^24^, ^25^ In our cohort, SND was strongly associated with advanced age and atrial fibrillation, similar to the general population.^26^, ^27^ Elderly patients, especially those with atrial fibrillation, may have unrecognized SND and are at increased risk of developing clinically significant bradyarrhythmia. In regards to the timing of pacemaker implantation for SND after an inferior myocardial infarction, a relatively early implantation may be reasonable considering the less reversible nature of SND, especially for older patients with atrial fibrillation and for those with significant bradyarrhythmia preventing beta‐blocker initiation. The relationship between SND and mortality is elusive since SND is predominantly seen in elderly patients with other comorbidities. Survival of patients with isolated SND (without structural heart disease) was comparable to the age‐ and sex‐matched population.^28^ In our cohort, although patients with SND had a higher proportion of death, SND was not associated with increased in‐hospital mortality, suggesting a limited causal impact of SND on in‐hospital mortality if any.

### 4.4 Study limitations

There are several limitations to our study inherent to any retrospective database analysis. For instance, the actual incidence of some conduction disorders may be underestimated in our results. This is particularly true for SND which often occurs transiently with a more insidious presentation than HDAVB. Overall, however, it is likely that clinically relevant conduction disorders were captured in the majority of patients. Since the NIS database does not contain the date of each diagnosis, we could not ascertain the time of occurrence and progression or regression of the conduction disorders. In addition, the nature of the database, derived from administrative coding, did not allow us to ascertain the exact indication or clinical rationale for each pacemaker implantation. Information about medications used such as beta‐blocker was not available in the database, although such medications may have contributed to conduction disorders as previously mentioned. Lastly, we only had in‐hospital data and were unable to evaluate post‐discharge conduction disturbances.

## 5 CONCLUSION

Our analysis of a large contemporary inpatient database showed that HDAVB and SND were observed in 4.0% and 0.6% of the patients with inferior STEMI, respectively. In patients with HDAVB, permanent pacemakers were implanted relatively early (the median interval of 3 days since admission) despite its highly reversible nature. Further studies are awaited to define the optimal waiting period before permanent pacemaker implantation in patients with conduction disorders following inferior STEMI.

## CONFLICT OF INTEREST

The authors declare no potential conflict of interests..

## Supporting information


**TABLE S1** Codes used to identify comorbidities and procedures
