# Supplementary material for: Contemporary practice pattern of permanent pacing for conduction disorders in inferior ST‐elevation myocardial infarction
Source: Clin Cardiol. 2019 Jun 7;42(8):728–34. doi: 10.1002/clc.23210 (PMC6671775; doi:10.1002/clc.23210)
Supplement: Supplementary file 1 — TABLE S1 Codes used to identify comorbidities and procedures [file CLC-42-728-s001.docx]

**Supplemental Table: Codes used to identify comorbidities and procedures**

|  | Source | Codes |
| --- | --- | --- |
| Hypertension | NIS | Elixhauser comorbidity |
| Diabetes | NIS | Elixhauser comorbidity |
| Dyslipidemia | CCS | CCS 53 |
| Current smoking | ICD-9-CM | 305.1 |
| Obesity | NIS | Elixhauser comorbidity |
| Coronary artery disease | CCS | CCS 101 |
| Prior myocardial infarction | ICD-9-CM | 412 |
| Prior PCI | ICD-9-CM | V45.82 |
| Prior CABG | ICD-9-CM | V45.81 |
| Prior stroke/TIA | ICD-9-CM | V12.54 |
| Congestive heart failure | CCS | CCS 108 |
| Anemia | NIS | Elixhauser comorbidity |
| Renal failure | NIS | Elixhauser comorbidity |
| Chronic pulmonary disease | NIS | Elixhauser comorbidity |
| Peripheral vascular disease | NIS | Elixhauser comorbidity |
| Atrial fibrillation or atrial flutter | ICD-9-CM | 427.31, 427.32 |
| **Clinical presentations** |  |  |
| Cardiac arrest | CCS | CCS 107 |
| Cardiogenic shock | ICD-9-CM | 785.51 |
| **In-hospital procedures** |  |  |
| PCI | ICD-9-CM | 36.01,36.02, 36.05, 36.06, 36.07, 00.66, and 17.55 |
| CABG | CCS | CCS 44 |
| Temporary pacemaker | ICD-9-CM | 37.78 |
| Permanent pacemaker | ICD-9-CM | 37.70, 37.71, 37.72, 37.73, 37.74, 37.80, 37.81, 37.82, 37.83, 00.50 |

CABG: coronary artery bypass grafting, CCS: Clinical Classifications Software, ICD-9-CM: International Classification of Diseases 9^th^ Edition, NIS: national inpatient sample, PCI: percutaneous coronary intervention, TIA: transient ischemic attack
